# The Moderating Role of Virtual Arm Embodiment for Upper Limb Rehabilitation in Stroke Patients with Proprioceptive Deficit: A Pilot Study

**DOI:** 10.3390/bs16071180

**Published:** 2026-07-13

**Authors:** Sara Ventura, Giada Lullini, Sara Castaldini, Cristina Russo, Stefano Triberti, Alessia Tessari

**Affiliations:** 1 Instituto Polibienestar, University of Valencia, Avenida Blasco Ibañez 21, 46010 Valencia, Spain; 2 Department of Psychology, University of Bologna, Viale Berti Pichat 5, 40126 Bologna, Italy; 3 UOC di Medicina Riabilitativa e Neuro-Riabilitazione, IRCCS Istituto delle Scienze Neurologiche di Bologna, Azienda USL Bologna, Via Altura, 3, 40139 Bologna, Italy; 4 LAHTI Laboratory for Advanced Human-Technology Interaction, Pegaso University, 20126 Milan, Italy; stefano.triberti@unipegaso.it; 5 Department of Psychology and Health Sciences, Pegaso University, 80143 Naples, Italy

**Keywords:** proprioceptive deficit, stroke, motor rehabilitation, embodiment, virtual reality

## Abstract

Stroke is the second leading cause of death worldwide and a major cause of long-term disability. These deficits may alter the body representation of the affected limb. Recent evidence suggests that Virtual Reality (VR), particularly when integrating body illusion paradigms, may enhance neuroplasticity and improve motor and proprioceptive abilities after stroke. This study investigated the effectiveness of a VR-based rehabilitation program for upper-limb motor and proprioceptive deficits. Participants were adults (both sexes) aged 18–85 years with ischemic or haemorrhagic stroke occurring from 2 to 18 months before recruitment, moderate to severe upper limb motor impairment (Motricity Index ≤ 80), altered proprioceptive function of the affected limb (failure on 3 of 4 Thumb Location Test trials), and the ability to understand and provide written informed consent. Eligible participants were undergoing a 4-week VR rehabilitation program and assessed at baseline and post-intervention. Twelve patients (four female) participated in this pilot study (Mage = 52.08, SDage = 16.03), with a mean time since stroke of 12 months. Non-parametric analyses showed significant improvements in motor abilities, including the Motricity Index (shoulder *p* = 0.039, elbow *p* = 0.024, pinch *p* = 0.011), Fugl–Meyer Assessment (*p* = 0.050), and Box and Block Test (*p* = 0.008). No significant improvement was observed in the Rubber Hand Illusion for the affected arm in terms of embodiment (*p* = 0.859). Regression analyses demonstrated that embodiment exerted a significant positive effect on post-intervention motor abilities (*p* = 0.004). In conclusion, VR-based rehabilitation may improve upper-limb motor performance after stroke modulates by proprioceptive ability, and that individual differences in embodiment may play an important role in modulating treatment-related motor outcomes.

## 1. Introduction

Stroke remains over years one of the leading causes of mortality and long-term disability worldwide. Its incidence increases with age, particularly after 55 years, with most cases occurring in individuals over 65 years old (Saini et al., 2021). Despite advances in acute medical management, stroke continues to produce severe functional consequences: mortality rates remain high during the first months after the event, and among survivors, only a minority achieve full recovery, whereas most require prolonged rehabilitation and may experience persistent disability. In this context, effective and accessible rehabilitation interventions are crucial to improve functional outcomes and quality of life (Duncan et al., 2005; Aprile et al., 2020; Singh et al., 2021; Feigin et al., 2025).

Upper limb impairment is among the most common and disabling consequences of stroke (Raghavan, 2015). Motor deficits frequently include muscle weakness, loss of dexterity, impaired reaching and grasping abilities, and reduced coordination, limiting the performance of activities of daily living such as eating, dressing, and personal hygiene. These limitations often increase dependence on caregivers and reduce autonomy (Pollock et al., 2014). Beyond motor dysfunction, stroke survivors often present sensorimotor and proprioceptive impairments that compromise the integration of sensory feedback and motor planning, further hindering functional recovery (Meyer et al., 2014).

From a neurocognitive perspective, damage to cortical and subcortical networks involved in movement execution and body-related processing may alter body representation and the sense of embodiment (Dijkerman & Lenggenhager, 2018, Tessari et al., 2021). In the present study, body representation is defined as the internal multisensory model of the body, integrating proprioceptive, sensory, and motor information to support body awareness, movement planning, and interaction with the environment. Embodiment can be considered the subjective experience arising from this body representation and includes components such as ownership, agency, and self-location (Carruthers, 2008). After stroke, altered proprioceptive and kinaesthetic processing may disrupt these components, leading to distorted perception of the affected limb and difficulties in integrating sensory and motor signals. In some cases, patients may experience reduced awareness of the affected limb or even develop delusional beliefs about limb ownership. Such disturbances in body representation may negatively affect motor performance and adaptation during rehabilitation (Pia et al., 2020; Matamala-Gomez et al., 2020; Lo et al., 2023).

Neuroplasticity, the brain’s capacity to reorganize structurally and functionally after injury, represents the neurobiological basis of post-stroke recovery (Alia et al., 2017). Rehabilitation interventions that promote multisensory integration and enhance body awareness may facilitate this process. Among these, body illusion paradigms have shown promise in modulating body representation and improving motor outcomes. Mirror therapy, for example, exploits visual feedback from the unaffected limb to induce the illusion of movement or ownership in the affected limb, potentially promoting cortical reorganization and functional gains (Thieme et al., 2013; Y. Zhang et al., 2022).

In recent years, Virtual Reality (VR) demonstrated its effectiveness in post-stroke rehabilitation: for example, a meta-analysis from 2023 on digital interventions for improving physical activity and reducing sedentary behavior in stroke patients reported 6 studies out of 16 employing VR (Wang & Kassavou, 2023). VR enables the creation of controlled, interactive, and customizable environments in which patients can perform task-oriented exercises with real-time feedback. Both immersive and non-immersive VR systems have demonstrated efficacy in improving upper limb motor recovery compared with conventional rehabilitation. Immersive VR, in particular, may enhance engagement, motivation, and the sense of presence, while also allowing the integration of body illusion paradigms through the visualization of a virtual limb synchronized with the patient’s movements (Holden et al., 2007; Kim et al., 2020). Recent evidence suggests that combining VR with embodiment-based interventions may strengthen neuroplastic mechanisms and improve motor recovery after stroke. Specifically, the induction of ownership over a virtual arm may act as a modulating factor in rehabilitation outcomes by facilitating the reorganization of body representation and sensorimotor integration (Turolla et al., 2013; Weber et al., 2019; Hsu et al., 2022). However, although the effectiveness of VR for motor rehabilitation is increasingly supported, fewer studies have specifically investigated its impact on proprioceptive deficits and embodiment-related processes.

Therefore, the present study aimed to evaluate the effectiveness of a virtual reality–based rehabilitation program in addressing upper limb motor and proprioceptive impairments following stroke. In addition, this study explored whether the achievement of embodiment toward the virtual arm may moderate rehabilitation outcomes. Based on the previous literature, we hypothesized that participants undergoing the VR intervention would show improvements in motor and proprioceptive abilities, and that stronger embodiment of the virtual limb would be associated with better rehabilitation outcomes.

## 2. Materials and Methods

### 2.1. Participants

Stroke patients were enrolled by the Neurorehabilitation Unit of the Institute of Neurological Sciences of Bologna. Recruitment was conducted between Fall 2022, following ethics approval, and January 2025. Eligible patients participated in a four-week Virtual Reality rehabilitation program and were assessed at three time points: screening (T0), pre-intervention (T1), and immediately after the intervention (T2). Patients respected the following inclusion criteria: (a) Be 18 to 85 years old; (b) Suffer from ischemic or haemorrhagic stroke; (c) The stroke event must have occurred from two to eighteen months before the recruitment; (d) Patients must have moderate to severe upper limb motor deficit established by a score of ≤80 on the Motricity Index, and the alteration of sensorimotor and proprioception abilities of the injury upper limb, established by the failure in 3 proofs up to 4 of the Thumb Location Test; (e) Patients must understand and sign the written consent for enrolment. Conversely, patients were excluded if they met at least one of the following criteria: (a) Severe psychiatric or behavioral disturbances (e.g., psychosis, severe depression, apathy, or marked psychomotor agitation), significant cognitive impairment, or a confusional state characterized by temporal and/or spatial disorientation observed during clinical conversation. When uncertainty was present, the presence of delirium/confusion was further screened using the 4AT (Bellelli et al., 2014); (b) Severe upper limb motor impairment, defined by Motricity Index scores below established cut-offs (grip < 11, elbow flexion < 14, shoulder abduction < 14; Bohannon, 1999); (c) Marked language comprehension deficits, indicated by a Token Test score < 2 (De Renzi & Vignolo, 1962); and (d) Severe spatial neglect, defined as a score > 3 on the Albert’s test (Albert, 1973).

### 2.2. Assessment

Table 1 summarizes the assessment protocol. Neuropsychological assessments were administered only at baseline (T0) to exclude cognitive deficits according to the inclusion criteria and to characterize the participants’ cognitive profile. Motor and proprioceptive assessments were administered before and after the rehabilitation program to evaluate changes in these functions following the intervention. The tests adopted were:

#### 2.2.1. Motor Assessment

- Fugl–Meyer (Platz et al., 2005). This was used to evaluate upper limb sensorimotor function. The scale includes 33 items rated on a 3-point ordinal scale (0–2), yielding a maximum score of 66, with higher scores indicating better motor recovery.
- Motricity Index (Bohannon, 1999). This is a test for upper limbs with scores ranging from 0 to 100. The test evaluated the shoulder abduction, the elbow flexion, and the “grip and pinch” abilities.
- Box and Blocks test (Mathiowetz et al., 1985). This contains 150 wooden cube blocks (1 inch). The participants were told to move one-by-one blocks as many as possible from a rectangular box container to the other of equal size within 60 s. Both hands’ scores of the BBT were calculated, respectively, by the number of blocks transferred.

#### 2.2.2. Proprioception Assessment

- Thumb Location Test (Rand, 2018). This evaluates the ability of individuals to accurately locate their thumbs without visual cues. During the test, the individual typically closes their eyes or is blindfolded, and the examiner moves the person’s thumb to different positions. The individual is then asked to indicate the location of their thumb by pointing to it with their other hand or verbally describing its position and it is scored from 0 = not accurate to 2 = completely accurate.
- Rubber Hand Illusion (Romano et al., 2021). This is a self-report questionnaire that evaluates the participants’ ability to perceive a rubber hand as his/her own in terms of ownership, location, and agency. The test is administered after the rubber hand induction by a professional with patient’s injury limb. The scale is score from −3 (not at all) to +3 (completely).

#### 2.2.3. Neuropsychological Assessment

- Short screening test for ideo-motor apraxia (STIMA) (Tessari et al., 2015). This was used to screen for ideomotor apraxia, a disorder characterized by impaired gesture imitation. The assessment includes two sets of gestures: meaningful (intransitive) actions and meaningless gestures, which are presented separately. Participants are asked to reproduce each gesture as accurately as possible. Performance is scored according to the number of attempts required for correct imitation, with higher scores reflecting the need for additional attempts.
- Raven progressive matrices (Carpenter et al., 1990) were administered to assess abstract reasoning abilities and non-verbal intellectual functioning. The test comprises a set of progressively more complex visual stimuli in which participants must infer the underlying relationships among elements and select the option that correctly completes the missing part of each matrix.
- Trials Making Test (A and B) (Tombaugh, 2004) to evaluate patient’s attention. This was administered to evaluate attentional processes and executive control. Part A assesses visual scanning and processing speed through a sequential number-connecting task, whereas Part B additionally requires set-shifting abilities by asking participants to alternate between numerical and alphabetical sequences. Faster completion times indicate better performance.
- The Attentional Matrices Test (Abbate et al., 2007) is a neuropsychological test used to assess selective and sustained attention, visual scanning, and processing speed. Participants are required to identify target numbers within structured matrices under time constraints, providing a measure of attentional efficiency and concentration abilities.
- Corsi Test (visuospatial) (Piccardi et al., 2013) was administered to evaluate visuospatial short-term memory and spatial span. Participants were required to reproduce a series of block sequences presented by the examiner, maintaining the same order of presentation. The task becomes progressively more challenging as the number of blocks within each sequence increases. The primary outcome measure is the longest sequence accurately reproduced, which reflects the individual’s visuospatial memory capacity.
- Monaco Test (or digit span forward and backward) (Monaco et al., 2013). This evaluates short-term memory (Digit Span Forward) and working memory capacity (Digit Span Backward). Participants were presented with sequences of digits and asked to reproduce them either in the same order (forward condition) or in reverse order (backward condition). Sequence length gradually increased, and administration was terminated following two consecutive incorrect responses at the same span level.
- Token Test (De Renzi & Vignolo, 1962) to evaluate the language comprehension. The task requires participants to execute verbally delivered commands by selecting and manipulating simple geometric tokens according to specific rules. The complexity of the instructions increases progressively across trials, incorporating different linguistic structures and syntactic relationships. Performance is evaluated on the basis of the accuracy with which participants understand and carry out the requested operations.
- Barrage (Albert, 1973) peripersonal neglect and measures the patient’s spatial and selective attention abilities.
- Visual Object and Space Perception (VOSP) battery (Quental et al., 2013) to evaluate distinct components of visual processing, including object recognition and spatial analysis. The object perception subtests measure the ability to identify, match, and discriminate visual stimuli, including degraded or fragmented figures. The spatial perception subtests assess higher-order visuospatial skills such as orientation judgment, spatial relationship processing, and pattern analysis within a visual field.

### 2.3. Apparatus

**Figure 1:**
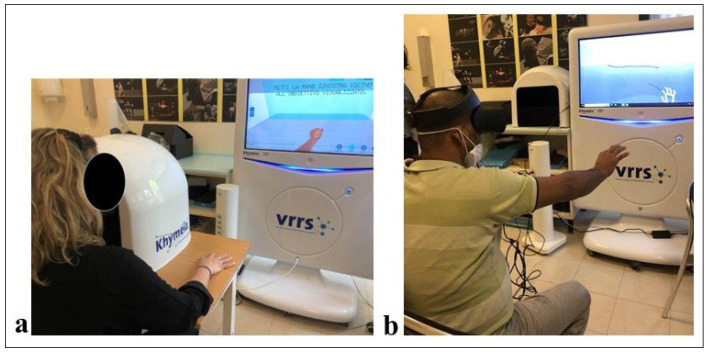
VR intervention: (**a**) Non-immersive Virtual Reality (HandBox); (**b**) Immersive Virtual Reality.

The systems adopted for the study was the Oculus Rift and the Virtual Reality Rehabilitation System-HandBox (Khymeia Group, Noventa Padovana, Italy) for the non-immersive session. The HandBox system consists of a dedicated enclosure in which patients place the upper limb undergoing rehabilitation. The device is equipped with a motion-tracking sensor that detects upper-limb movements and reproduces them in real time on a display, allowing patients to receive continuous visual feedback during task execution. The VRRS (Virtual Reality Rehabilitation System) display is a 30-inch screen that serves as the central hub of the rehabilitation platform. Through USB connectivity, it can be integrated with a range of specialized peripheral devices designed to support different rehabilitation modules and therapeutic exercises. The system enables the delivery of personalized rehabilitation programs targeting various motor functions (Figure 1a). For the immersive session, an Oculus Rift head-mounted display was connected to the VRRS platform to provide immersive virtual reality training. The Oculus Rift features a stereoscopic display with head-tracking technology, allowing users to interact with a three-dimensional virtual environment through natural head movements. This immersive setup enhances the sense of presence and engagement during rehabilitation exercises (Figure 1b).

### 2.4. Intervention

The intervention included 12 one-hour sessions over four weeks (three per week), conducted in a quiet room at Bellaria Hospital in Bologna. Each session combined immersive and non-immersive VR rehabilitation. In immersive VR, patients sat wearing a head-mounted display and performed supervised exergames involving hand movements, such as object manipulation, positioning, and drawing tasks (Figure 1a). In the non-immersive condition, patients used the HandBox system, which tracks hand movements without sensors and displays them on a screen placed 1.5 m away (Figure 1b). Exercise difficulty was individualized according to the patient’s baseline motor performance, as assessed by the Motricity Index and the physiotherapist’s clinical evaluation. The VRRS allows task difficulty to be adjusted across ten levels (1–10), with higher levels requiring greater movement precision, range of motion, speed, coordination, and task complexity. In addition, sensor sensitivity parameters were calibrated to match the patient’s residual motor function, enabling the system to detect even minimal voluntary movements. Patients with more severe impairments started at lower difficulty levels and higher sensor sensitivity settings, whereas patients with better motor abilities performed more demanding tasks with lower sensitivity thresholds. Throughout the intervention, the physiotherapist monitored performance and progressively adjusted difficulty and sensitivity settings to maintain an appropriate challenge level while ensuring successful task completion. Each session included 30 min of immersive and 30 min of non-immersive training, with continuous physiotherapist supervision. Exercise difficulty and sensor sensitivity were personalized (levels 1–10) based on baseline motor function, allowing even patients with minimal hand ability to perform tasks.

## 3. Data Analysis

All variables were analyzed using the Statistical Package for the Social Sciences (SPSS) version 11.5 (SPSS Inc., Chicago, IL, USA) and Jasp. As the data were not normally distributed, non-parametric tests were conducted. Given the non-normal distribution of the data, changes between baseline and post-intervention were analyzed using the Wilcoxon signed-rank test. Furthermore, a moderation analysis was performed to investigate whether virtual arm embodiment moderates the relationship between baseline and post-intervention motor performance. In each model, baseline performance was entered as the predictor, post-intervention as the dependent variable, and embodiment as the moderator.

## 4. Results

Twelve patients took part at the pilot study, four female and eight males, eight with their right and four with their left side affected by stroke. The mean age of all participants was 52.08 years (range 19–75 years) and the mean time since stroke was 12 months (range 2–34 months). The pre-intervention range for all participants on the upper limb Motricity Index (total) was 40–93 points, Thumb Location was 0.92 (range −3–3) and the Fugl–Meyer total was 58–124 points. Furthermore, patients demonstrated no cognitive impairments in language, memory, and attention abilities and no signs of unilateral spatial neglect emerged, as all patients scored within the normal range on the cancelation (barrage) test. Baseline characteristics are presented in Table 2.

### 4.1. Difference on Time on Motor Abilities

**Figure 2:**
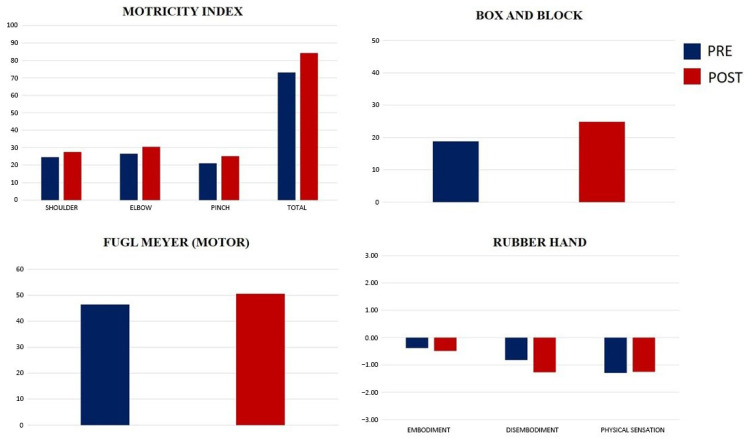
Motor outcomes measure scores at each time point.

Results of non-parametric analysis demonstrated a significant improvement in motor abilities (Figure 2 and Table 3): Motricity Index (shoulder Z = −2.060, *p* = 0.039, *d* = 0.40, elbow Z = −2.264 *p* = 0.024 *d* = 0.44, pinch Z = −2.549 *p* = 0.011 *d* = 0.66, and total score Z = −2.937 *p* = 0.003 *d* = 0.66); Fugl–Meyer total score on motor abilities including shoulder, wrist, hand and coordination and speed (Z = −1.957 *p* = 0.050, *d* = 0.48); Box and Block (Z = −2.670 *p* = 0.008, *d* = 0.55 ). However, patients did not improve in Rubber Hand Illusion of their injury arm: Embodiment (Z = −0.178 *p* = 0.859, *d* = 0.74), Disembodiment (Z = −0.770 *p* = 0.441, *d* = 0.91), Physical sensations (Z = −0.079 *p* = 0.937, *d* = 0.17).

### 4.2. Embodied the Virtual Arm Moderates Change on Motor Abilities

A moderation analysis was conducted to examine whether virtual arm embodiment influenced the relationship between baseline (T0) and post-intervention (T1) motor performance assessed by the Motricity Index (Total), Fugl–Meyer and Box and Blocks Tests.

Moderation analyses revealed that embodiment was significantly associated with post-intervention motor performance and significantly moderated the relationship between baseline (T0) and post-intervention (T1) motor outcomes.

**Figure 3:**
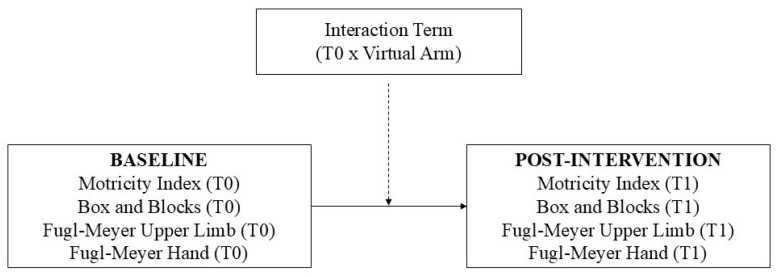
Conceptual diagram of the moderation analysis.

Results demonstrated that Embodiment exerted a significant positive effect on post-intervention motor abilities for Box and Blocks (*β* = 14.319, *SE* = 5.008, *z* = 2.859, *p* = 0.004), and the interaction between baseline performance and embodiment was statistically significant (*β* = −0.394, *SE* = 0.191, *z* = −2.069, *p* = 0.039), indicating a moderation effect; for Motricity Index (Total) (*β* = 50.167, *SE* = 16.197, *z* = 3.097, *p* = 0.002), and the interaction between baseline performance and embodiment was statistically significant (*β* = −0.674, *SE* = 0.205, *z* = −3.291, *p* ≤ 0.001). Finally, the Embodiment exerted a significant positive effect on post-intervention motor abilities for Fugl–Meyer upper limb (*β* = 10.858, *SE* = 4.413, *z* = 2.460, *p* = 0.014), interaction between baseline performance and embodiment was also statistically significant (*β* = −0.325 *SE* = 0.153, *z* = −2.121, *p* = 0.034), and for Fugl–Meyer hand (*β* = 7.880, *SE* = 1.680, *z* = 4.690, *p* ≤ 0.001), and the interaction between baseline performance and embodiment was statistically significant (*β* = −0.731, *SE* = 0.175, *z* = −4.185, *p* ≤ 0.001). Finally, the higher levels of virtual arm embodiment were associated with improved motor outcomes (Figure 3).

## 5. Discussion

In our study, patients with upper limb motor and proprioceptive deficits after stroke undergoing VR-based rehabilitation demonstrated meaningful improvements in upper-limb motor abilities following the four-week intervention. Virtual arm embodiment may represent a potential moderator of rehabilitation outcomes, as a stronger sense of ownership over the virtual limb could enhance engagement, sensorimotor integration, and treatment efficacy. Improvements were observed across several aspects of motor function, including strength, manual dexterity, and functional arm use, suggesting a broad positive impact of the intervention on motor recovery. These findings are consistent with an increasing body of evidence supporting the potential of virtual reality-based rehabilitation to enhance motor outcomes after stroke (Jack et al., 2001; Saposnik et al., 2011; Hao et al., 2024; Laver et al., 2025).

Significant improvements in the Motricity Index indicate enhanced proximal and distal muscle strength, including shoulder abduction, elbow flexion, and pinch function. These domains are critical for functional reaching and grasping activities and represent common targets of upper limb rehabilitation. Similarly, the improvement in the Fugl–Meyer Assessment suggests a broader recovery of motor control, coordination, and upper limb synergies. Improvement in the Box and Block Test further indicates enhanced manual dexterity and gross motor manipulation abilities. Taken together, these results support the notion that repeated, task-oriented, multisensory training delivered through VR may facilitate functional motor recovery (Langhorne et al., 2009). Several mechanisms may explain these improvements.

First, VR provides intensive and repetitive practice, which is known to drive use-dependent neuroplasticity. Repetition of goal-directed movements strengthens sensorimotor pathways and may contribute to cortical reorganization after stroke (Kim et al., 2020). Second, VR environments provide real-time augmented visual feedback, allowing patients to continuously monitor and correct movement execution. Such feedback may compensate for impaired proprioception and enhance sensorimotor integration (Sousa et al., 2016). Third, the gamified and immersive nature of VR may increase motivation, engagement, and adherence to rehabilitation programs, factors known to influence treatment effectiveness (Kim et al., 2020). In the present study, the combination of immersive and non-immersive VR may have maximized these benefits by integrating ecological motor tasks with highly engaging interactive experiences.

Despite the significant improvement in motor abilities, no significant changes in embodiment scores were found after the induction of the arm illusion using the Rubber Hand. This finding is in line with the previous literature showing the heterogeneous effects of body ownership manipulations in post-stroke and general populations. For instance, Burin et al. (2015) reported that hemiplegic patients can experience the illusion, although its intensity may not directly relate to motor recovery. Moreover, ownership-related measures may dissociate from proprioceptive processes (Rohde et al., 2011), and embodiment itself is highly sensitive to contextual and visual features of the illusion paradigm (Pyasik et al., 2020).

A particularly novel finding of this study concerns the role of embodiment in modulating rehabilitation outcomes. Although no significant changes emerged in explicit embodiment ratings toward the affected limb as measured by the Rubber Hand Illusion, moderation analyses revealed that the degree of embodiment experienced toward the virtual arm significantly influenced motor recovery. Specifically, stronger embodiment was associated with better performance in global motor function, hand-specific function, and manual dexterity. The present finding is in accordance with a published literature review, which investigates the modulating role of embodiment for motor rehabilitation after stroke (Ventura et al., 2023). The reviewed studies demonstrated that inducing a body ownership illusion through VR could facilitate motor rehabilitation for the upper injured limb (Perez-Marcos et al., 2017; Cha et al., 2021; Fregna et al., 2022). This effect may be driven by synchronized visuo-sensory stimulation between the patient’s physical body and the virtual body, which may modulate cognitive processing and facilitate rehabilitation (Kilteni et al., 2013). Tambone et al. (2021) also found that the body illusion was a positive predictor of motor rehabilitation: patients who perceived the body avatar as their own body improved their gait and balance abilities better than those who did not reach the illusion (Tambone et al., 2021). From a neurocognitive perspective, embodiment of a virtual limb may facilitate the integration of visual, proprioceptive, and motor signals, thereby strengthening the internal model of the affected limb. This process may improve motor planning and execution by reducing discrepancies between predicted and actual sensory feedback (Matamala-Gomez et al., 2020). Moreover, experiencing ownership over a virtual arm capable of movement may activate motor-related cortical networks through mechanisms similar to motor imagery, action observation, and mirror therapy. Such activation may be particularly relevant in patients with impaired voluntary movement, as it may promote adaptive plasticity even in the absence of complete motor execution (J. J. Zhang et al., 2018; Errante et al., 2022). The absence of significant changes in Rubber Hand Illusion scores may appear inconsistent with the moderation findings; however, several explanations are possible. First, explicit embodiment questionnaires may lack sensitivity to capture subtle or dynamic changes in embodiment over time, especially in clinical populations with altered proprioceptive awareness. Embodiment is a multidimensional and context-dependent construct that may fluctuate during interaction with the VR system rather than being reflected in static post-task measures. Future studies should include implicit or phenomenological measures to assess proprioceptive processing and embodiment of the affected arm, as self-report measures may be limited by patients’ ability to fully understand and accurately report their subjective experiences (Zepeda et al., 2025). Second, the Rubber Hand Illusion assesses embodiment toward the affected physical limb, whereas the moderation analysis focused on embodiment of the virtual arm during rehabilitation. These constructs, while related, may reflect participants’ confusion from virtual to rubber hand. Finally, the small sample size may have limited statistical power to detect longitudinal changes in embodiment.

These findings have important clinical implications. If embodiment acts as a significant moderator of rehabilitation outcomes, future VR interventions should not only provide repetitive motor training but also explicitly optimize embodiment-related features. For example, increasing visuomotor synchrony, improving avatar personalization considering the severity of patients’ motor deficits, enhancing visuo-proprioceptive congruency, and integrating haptic or tactile feedback may strengthen ownership and agency over the virtual limb. Such design strategies could maximize the therapeutic effectiveness of VR systems and pave the way toward more personalized neurorehabilitation approaches.

Despite these promising results, several limitations should be acknowledged. First, the sample size was small, limiting statistical power and generalizability. Given the very small sample size and the high heterogeneity of the participants, the moderation analysis should be interpreted with caution, as the study was likely underpowered to detect reliable interaction effects, and the results are therefore considered exploratory rather than confirmatory. Second, the absence of a treatment-as-usual control group in the present analysis prevents firm conclusions regarding the superiority of VR over conventional rehabilitation. Third, the sample was heterogeneous in terms of age, time since stroke, and severity of impairment, factors that may influence responsiveness to treatment. Fourth, follow-up assessments were not conducted, preventing conclusions regarding the long-term maintenance of gains. Future research should address these limitations by conducting adequately powered randomized controlled trials comparing VR-based embodiment interventions with standard rehabilitation or non-embodied VR protocols. Longitudinal designs with follow-up assessments are needed to evaluate the durability of treatment effects. Furthermore, mechanistic studies combining behavioral outcomes with implicit techniques could clarify the neural substrates through which embodiment influences recovery. Understanding which patient characteristics predict responsiveness to embodiment-based rehabilitation may also help identify subgroups most likely to benefit from such interventions.

## 6. Conclusions

In conclusion, this pilot study provides preliminary evidence that VR-based rehabilitation may improve both upper limb motor and proprioceptive outcomes after stroke. Importantly, our findings suggest that embodiment of the virtual arm may play a relevant role in modulating these effects. These results support the emerging perspective that the therapeutic potential of VR in neurorehabilitation extends beyond repetitive motor practice and may involve mechanisms related to body representation and sensorimotor integration. Overall, this study lays the groundwork for the development of more personalized VR-based rehabilitation interventions for stroke survivors.

## Figures and Tables

**Table 1:** Baseline and post-intervention assessments.

Patients’ Abilities	Test	T0	T1
Motor	Fugl–Meyer (Platz et al., 2005)	x	x
	Motricity Index (Bohannon, 1999)	x	x
	Box and Blocks (Mathiowetz et al., 1985)	x	x
Proprioception	Thumb Location Test (Rand, 2018)	x	x
	Rubber Hand Illusion (Romano et al., 2021)	x	x
Apraxia	Short screening test for ideo-motor apraxia (STIMA) (Tessari et al., 2015)	x	
Intelligence	Raven progressive matrices (Carpenter et al., 1990)	x	
Attention	Trials Making Test (A and B) (Tombaugh, 2004) Attentional Matrices (Abbate et al., 2007)	x	
Memory	Corsi Test (visuospatial) (Piccardi et al., 2013)	x	
	Monaco Test (span forward and backward) (Monaco et al., 2013)	x	
Language comprehension	Token Test (De Renzi & Vignolo, 1962)	x	
Visuo-spatial	Barrage (Albert, 1973)		
	Visual object and space perception (VOSP) (Quental et al., 2013)	x	

Note: T0 refers to the test administered before the assessment, whereas T1 refers to the test administered after the assessment.

**Table 2:** Patients’ baseline characteristics.

	M (SD) N = 12	Median	IQR
Clinical Variables			
Age (years)	52.08 (16.03)	58	18
Sex			
Female	4		
Male	8		
Time since stroke (months)	12 (11)	6.50	14.25
Side most affected			
Right hemiplegia	8		
Left hemiplegia	4		
Neuropsychological Variables			
Token Test	3 (1.28) *		
Stima	3.92 (0.29) *		
Raven	2.67 (1.44) *		
Attentional Matrices	2.08 (1.38) *		
Trial Making Test A	2.83 (1.12) *		
Trial Making Test B	2.58 (0.99) *		
Corsi	2.92 (1.44) *		
Digit Span Forward	2.83 (1.11) *		
Digit Span Backward	2.92 (1.38) *		
Vosp Shape Detection	19.58 (0.79)		
Vosp Object Decision	15.08 (3.05)		
Barrage	20 (0)		
Proprioception			
Thumb Location	0.92 (1.50)		

Note: * scores adjusted according to normative data (Equivalent Scores, range 0–4).

**Table 3:** Average and standard deviation on motor outcomes measure scores at each time point.

	Pre-Intervention M (SD)	Post-Intervention M (SD)
Motricity Index		
Shoulder	24.58 (5.80)	27.58 (6.47)
Elbow	26.50 (6.97)	30.50 (4.76)
Pinch	21.08 (6.29)	25.17 (9.10)
Fugl–Meyer		
Motor Function (A–D)	46.50/66 (13.89)	50.58 (12.66)
A. Upper Limbs	27.08/36 (7.80)	30.92 (5.48)
B. Wrist	7.92/10 (2.02)	8.08 (1.83)
C. Hand	8.33/14 (3.65)	9.92 (3.60)
D. Coordination and Speed	3.42/6 (1.73)	4.17 (1.75)
Sensory Function	8.42/12 (3)	9.17 (2.66)
Pain	19.33/24 (5)	20.75 (4.37)
Joint Function	20.92/24 (3.20)	21.33 (2.54)
Box and Block	18.83 (11.82)	24.83 (14.72)
Rubber Hand		
Embodiment	−0.38 (1.88)	−0.48 (2.03)
Disembodiment	−0.82 (1.61)	−1.26 (1.79)
Physical sensations	−1.29 (.83)	−1.25 (1.28)

## Data Availability

Dataset is available upon request on authors.
